# Comparison of unilateral biportal endoscopic decompression and percutaneous endoscopic lumbar decompression for single-segment degenerative lumbar spinal stenosis: a retrospective comparative study involving clinical efficacy and cost analysis

**DOI:** 10.3389/fmed.2026.1978780

**Published:** 2026-10-26

**Authors:** Yu Zhang, Jidong Ju, Jinchun Wu

**Affiliations:** Department of Orthopaedics, Jingjiang People’s Hospital Affiliated to Yangzhou University, Jingjiang, Jiangsu, China

**Keywords:** complications, cost, efficacy, lumbar spinal stenosis, unilateral biportal endoscopic decompression

## Abstract

**Objective:**

To compare the clinical efficacy and hospitalization medical cost associated with unilateral biportal endoscopic decompression (UBED) vs. percutaneous endoscopic lumbar decompression (PELD) for the treatment of single-segment degenerative lumbar spinal stenosis (DLSS).

**Methods:**

This retrospective study included 94 patients with single-segment DLSS, who received surgery between January 2023 and July 2025: 42 in UBED group and 52 in PELD group. Perioperative parameters, including intraoperative blood loss, fluoroscopy times, operative duration, time to postoperative ambulation, and length of hospital stay, were compared between groups. Additionally, patient-reported outcomes, namely, pain intensity (assessed using visual analog scale, VAS), functional disability (Oswestry disability index, ODI), and neurological function (Japanese Orthopaedic Association, JOA score), were evaluated preoperatively and at follow-up. Longitudinal between-group comparisons were performed using generalized estimating equations (GEE), with preoperative baseline scores as covariates and Bonferroni correction for *post-hoc* comparisons. Total and partial hospitalization costs were also compared. A generalized linear model (GLM) was employed to identify independent predictors of total hospitalization cost.

**Results:**

With regard to perioperative parameters, intraoperative blood loss was significantly greater in UBED group than in PELD group, whereas fluoroscopy times were significantly lower in UBED group (*p* < 0.05). No significant intergroup differences were detected in operative time, time to postoperative ambulation, or length of hospital stay (*p* > 0.05). Both groups showed significant improvements in ODI, VAS, and JOA score from baseline at all follow-up points (*p* < 0.05). In GEE analysis, UBED group showed lower VAS score for leg pain at 1 month, lower ODI at 1 and 6 months, and higher JOA score at 1 month (*p* < 0.001). Excellent-good rate was 97.6% in UBED group, significantly higher than that in PELD group (*p* = 0.039). Complication rate was significantly lower in UBED group (4.76%) than in PELD group (19.23%; *p* = 0.037). Total hospitalization cost was higher in UBED group than that in PELD group (17.46 vs. 15.82 × 10^3^RMB, *p* = 0.012). Multivariate regression analysis by GLM confirmed surgical approach, length of hospitalization, and intraoperative bleeding were independent predictors of total hospitalization cost.

**Conclusion:**

Both UBED and PELD are safe and effective minimally invasive approaches for single-segment DLSS. UBED was associated with slightly better early functional outcomes at 1 month and a numerically lower complication rate, but at a higher hospitalization cost. However, PELD was associated with lower hospitalization cost. In clinical practice, the selection of operative strategy should be individualized, with consideration given to both the patient's clinical characteristics and socioeconomic circumstances, to optimize the balance between therapeutic benefit and healthcare resource utilization.

## Introduction

Degenerative lumbar spinal stenosis (DLSS) is a degenerative spinal disorder characterized by pathological alterations, including osteophytic overgrowth, ligamentum flavum hypertrophy, and intervertebral disc protrusion, within lumbar spinal canal or neural foramina. These changes lead to narrowing of the canal or foramina, resulting in compression of the cauda equina or nerve roots (1). Clinically, hallmark manifestations include low back pain, radicular pain radiating into lower extremities, and neurogenic claudication. In advanced cases, patients may develop urinary and fecal dysfunction, as well as progressive weakness in the lower limb musculature. As a prevalent condition among older adults, the incidence of DLSS rises steadily with global population aging. It represents a leading cause of lower extremity disability and impaired quality of life in the elderly and imposes substantial economic and resource burdens on healthcare systems worldwide. Current management strategies for DLSS are broadly categorized into conservative and surgical approaches. Conservative therapy, comprising pharmacologic analgesia, physical therapy, rehabilitation exercises, and epidural steroid injections, is the initial treatment of choice for patients with mild-to-moderate symptoms. Surgical decompression is indicated for patients who fail to benefit from at least three months of standardized conservative management, exhibit progressive neurological deterioration, or demonstrate objective evidence of neural compromise (2).

Throughout the evolution of spinal surgery, traditional open decompression has long served as gold standard for surgical intervention in DLSS (3). Open decompression surgeries, including total and partial laminectomy for decompression, enable direct, full visualization of the spinal canal anatomy, allowing complete resection of hypertrophic osteophytes, thickened ligamentum flavum, and herniated intervertebral disc. These procedures achieve comprehensive decompression of the central canal, lateral recesses, and intervertebral foramina, and are associated with broad clinical indications. They remain clinically valuable, particularly in patients with lumbar instability, severe calcification, or complex, multilevel stenosis. However, open decompression carries substantial limitations. For example, extensive dissection of paravertebral musculature and ligamentous structures is required, resulting in significant iatrogenic injury to posterior spinal soft tissues, considerable intraoperative blood loss, pronounced postoperative incisional pain, and prolonged periods of bed rest and hospitalization. Furthermore, extensive bony resection may compromise spinal biomechanical stability, potentially leading to long-term sequelae such as postoperative spinal instability and accelerated degeneration of adjacent segments (4). In elderly patients with multiple comorbidities, the systemic physiological stress induced by surgical trauma further elevates perioperative risk (5).

To mitigate the morbidity associated with open surgery, spinal minimally invasive techniques have evolved continuously. Among these, percutaneous endoscopic lumbar decompression (PELD) has emerged as a widely adopted, evidence-based minimally invasive approach (6). PELD establishes a single working channel via a transforaminal trajectory, enabling targeted, endoscopically guided decompression. Crucially, it avoids extensive paravertebral muscle dissection and preserves spinal bony structure. Its key advantages include reduced surgical trauma, minimal blood loss, attenuated postoperative pain, and expedited functional recovery (7). Most patients can undergo the procedure under local anesthesia, thereby significantly reducing anesthetic risks and the incidence of perioperative complications, while also enabling shorter hospital stays and more rapid return to daily activities and work (8). Nevertheless, single-channel percutaneous endoscopy is inherently constrained by several limitations that restrict its broader clinical application. Specifically, the single working channel serves both as an observation conduit and a surgical instrument pathway, resulting in a narrow operative field and restricted instrument maneuverability. Consequently, achieving adequate and thorough decompression remains challenging in cases of severe central stenosis, extensive ligamentum flavum calcification, or widespread lateral recess stenosis. As a result, postoperative symptom relief may be incomplete, and the risk of symptom recurrence is comparatively higher. Moreover, this technique entails a steep learning curve and surgeons necessitate substantial time and experience to attain proficiency, particularly in managing complex cases (9). Intraoperative nerve injury risk is likewise strongly correlated with the operator's level of expertise.

To overcome the constraints of single-channel percutaneous endoscopy, particularly regarding limited working space and insufficient decompression capacity, unilateral biportal endoscopic decompression (UBED) has emerged as an innovative alternative and is increasingly being adopted in clinical practice (10). This approach employs two independent, dedicated channels: one for endoscopic visualization and the other for surgical instrumentation, thereby functionally separating real-time image monitoring from therapeutic intervention. It preserves the hallmark advantage of minimally invasive techniques, minimal soft-tissue disruption, while markedly expanding surgical freedom and improving visualization (11). Surgeons may utilize conventional open-surgical instruments through the work channel, facilitating more extensive and comprehensive decompression of the central canal, bilateral lateral recesses, and intervertebral foramina. The resulting decompression efficacy closely approximates that achieved with traditional open surgery. Furthermore, the learning curve associated with UBED is comparatively less demanding (12). The indications for UBED are broader, enabling effective management of complex cases, such as calcified stenosis and severe spinal canal stenosis, that are often beyond the scope of single-channel endoscopic techniques (13). Nevertheless, the current clinical adoption of UBED approach faces several practical challenges. UBED requires more sophisticated endoscopic equipment and specialized consumables, and the associated surgical instrumentation costs are substantially higher than those of PELD, thereby directly increasing overall patient medical expenditures (9, 14). Moreover, high-quality evidence regarding its long-term clinical efficacy, complication rates, and cost-effectiveness remains relatively scarce.

Currently, both UBED and PELD have been routinely incorporated into the surgical management of DLSS (15, 16). However, comparative studies evaluating their clinical efficacy have yielded inconsistent conclusions. Some investigations reported that UBED achieved more complete neural decompression and superior medium- to long-term functional outcomes (17), whereas others found no statistically significant difference in short-term functional recovery between both approaches (18, 19). Notably, findings vary across institutions, reflecting heterogeneity in surgical protocols, patient selection, and outcome assessment methodologies. Furthermore, the majority of existing studies focus exclusively on clinical outcomes and complication, but few undertake comprehensive health economic evaluations, including detailed analyses of procedural costs, resource utilization, and cost-effectiveness, to directly compare these two modalities. Consequently, robust, evidence-based guidance for optimal surgical decision-making and efficient allocation of healthcare resources remains limited.

Based on this background, our study retrospectively analyzed clinical data from patients with single-segment DLSS who received either of two minimally invasive procedures at a single center. Specifically, it compared perioperative parameters, clinical functional outcomes, postoperative complication rates, and hospitalization cost between UBED and PELD. The primary objective was to systematically evaluate clinical efficacy and hospitalization cost of both procedures, thereby providing evidence-based guidance for individualized surgical decision-making and rational allocation of healthcare resources in clinical practice, as well as practical insights to optimize minimally invasive treatment strategies for DLSS.

## Method

This was a retrospective comparative study conducted at Jingjiang People's Hospital. A retrospective review was performed on 94 consecutive patients diagnosed with single-level DLSS who received surgical intervention between January 2023 and July 2025. Patients were stratified into two cohorts based on surgical techniques: UBED group (*n* = 42, Figure 1A) and PELD group (*n* = 52, Figure 1B). Eligible patients were retrospectively identified from the institutional electronic medical record database according to predefined selection criteria.

**Figure 1:**
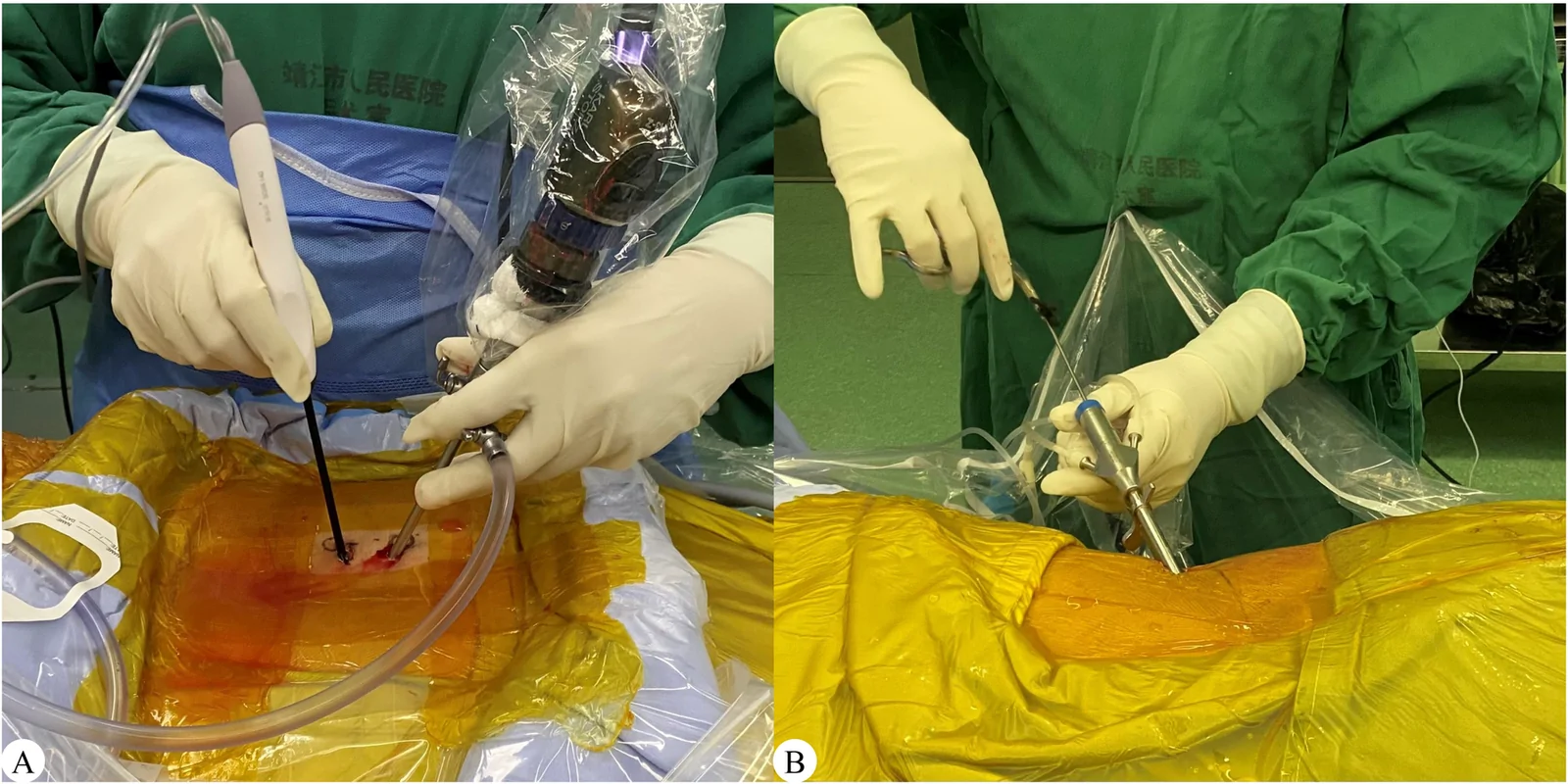
Operation schematic diagram of unilateral biportal endoscopic decompression **(A)** and percutaneous endoscopic lumbar decompression **(B)**.

Inclusion criteria were:

1. Diagnosis of DLSS confirmed by characteristic clinical manifestations, including neurogenic claudication, low back pain, and/or radicular symptoms such as leg numbness or weakness, supported by physical examination findings and definitive imaging evidence (CT and/or MRI);
2. Radiographically confirmed single-segment DLSS;
3. Failure of standardized conservative management, including pharmacotherapy, physical therapy, and rehabilitation;
4. Willingness and ability to provide informed consent and comply with scheduled follow-up assessments.

Exclusion criteria included:

1. Previous experience of lumbar surgeries;
2. Lumbar spondylolisthesis, instability, or deformity;
3. Lumbar tumors, tuberculosis, or fractures;
4. Severe diseases affecting anesthesia or surgery;
5. Follow-up duration less than 12 months.

### Surgical procedure

The choice of surgical method (UBED and PELD) was determined via collaborative decision-making, with consideration given to anatomical feasibility and patient preference. Both methods are considered technically applicable for single-segment DLSS. All procedures were performed by a single senior spine surgeon with more than 5 years of experience in spinal endoscopic surgery. Figure 2 illustrates the surgical procedure of UBED. Supplementary File 1 provide detailed information about the surgical procedures.

**Figure 2:**
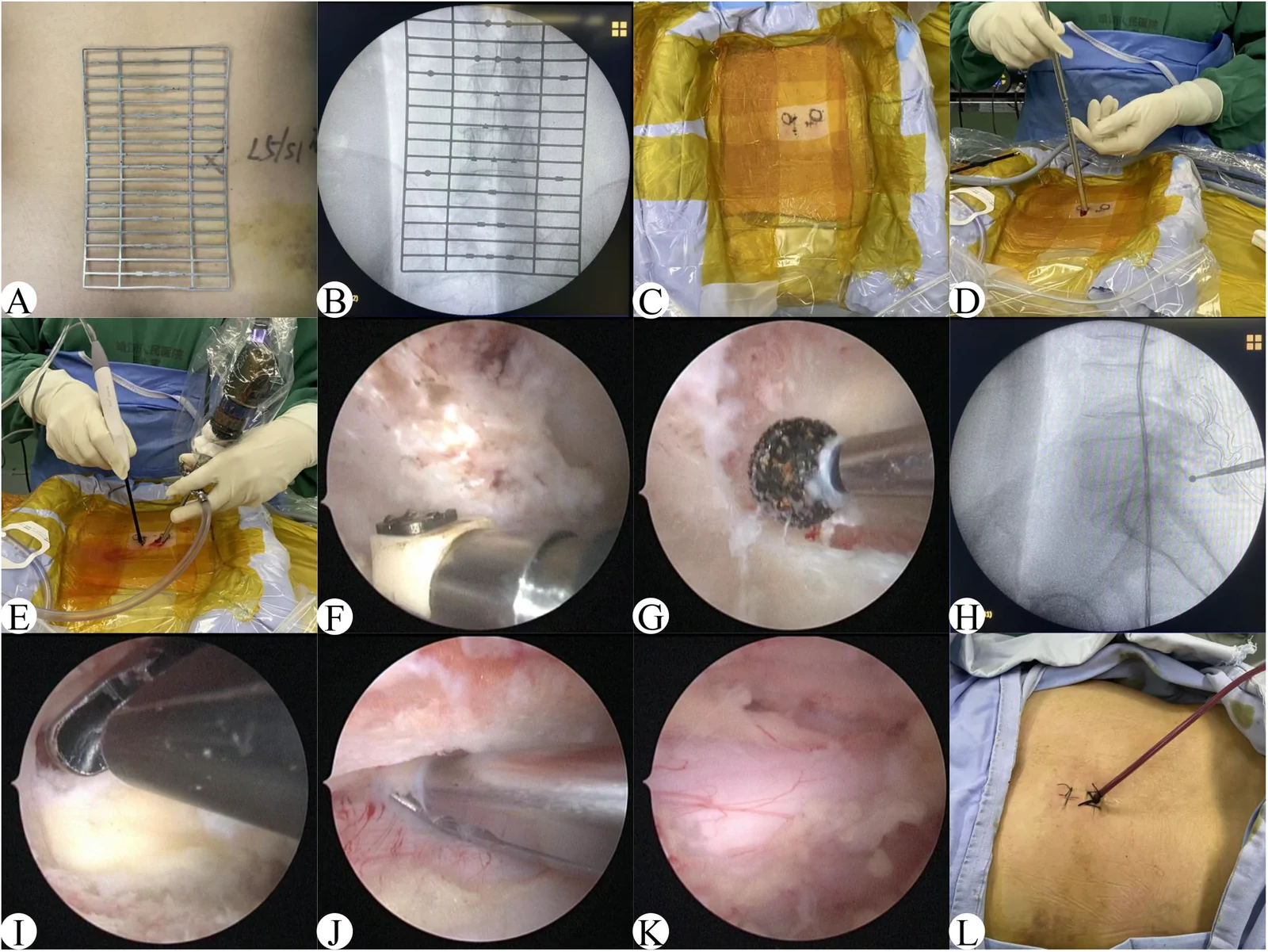
Surgical process of unilateral biportal endoscopic decompression. **(A–C)** Through fluoroscopy, a grid-like metal marker is used to mark the skin surface; **(D)** multiple cannulas are used to successively expand the soft tissue; **(E)** the surgeon uses both hands to operate the electrocoagulator and the endoscope simultaneously; **(F)** radiofrequency electrodes are used to form pathways through the intermuscular layer; **(G)** a high-speed drill is used to remove part of the lamina; **(H)** using fluoroscopy, the surgical segment is reconfirmed; **(I,J)** the ligamentum flavum is removed; **(K)** after complete decompression, the relaxed dura mater and nerve roots are exposed; **(L)** a drainage tube is placed, and the incision is sutured.

### Data collection

Data were collected retrospectively from the preoperative period to the final follow-up visit. Patient demographics and perioperative parameters were extracted and analyzed from comprehensive medical record reviews. Demographic variables included age, sex, body mass index (BMI), duration of symptoms, comorbidities, and surgical segments. Spinal stenosis type was evaluated by independent spinal surgeons based on preoperative MRI and CT examinations. Perioperative variables encompassed operative duration, intraoperative blood loss, intraoperative fluoroscopy times, time to postoperative ambulation, and length of hospital stay, both before and after surgery.

Functional outcomes: Pain was evaluated via visual analogue scale (VAS; range 0–10) for both back and leg pain. Functional outcome was assessed according to Oswestry disability index (ODI; range 0%–100%) and Japanese Orthopaedic Association (JOA) scores (range 0–29). Outcomes was assessed preoperatively and at 3 days, 1 month, 6 months, and 12 months postoperatively.

Clinical efficacy: At 12 months postoperatively, clinical efficacy was classified according to modified MacNab criteria as follows: excellent (no pain or functional limitations; full return to work and daily activities), good (marked improvement with only mild residual symptoms), fair (partial improvement but persistent functional impairment), or poor (no improvement or clinical deterioration). Excellent–good rate was calculated as: (number of excellent cases + number of good cases)/total number of cases × 100%.

Furthermore, complications and reoperations were systematically documented. Total hospitalization costs and their components, including surgical expense, anesthesia expense, material cost, and medication cost, were extracted from the hospital billing records. All costs are presented in Renminbi (RMB) at actual hospital billing prices during the study period. No inflation adjustment was applied.

### Statistical analysis

All statistical analyses were performed using SPSS 26.0 software (IBM Corp., Armonk, NY, USA). Continuous variables were first assessed for normality using Shapiro–Wilk test. Normally distributed variables are presented as mean ± standard deviation, with between-group comparisons performed using independent-samples *t*-test. Variables that deviated from normality are presented as median (interquartile range, IQR), with between-group comparisons performed using Mann–Whitney *U* test. Within-group comparisons employed paired *t*-tests or Wilcoxon signed-rank tests, as appropriate. Categorical variables are expressed as frequencies and percentages, with between-group comparisons performed using chi-square test or Fisher’s exact test, as appropriate.

For repeated-measures outcomes, a generalized estimating equation (GEE) was adopted to account for within-subject correlation among repeated observations over time and to avoid inflation of type I error associated with multiple pairwise comparisons across time points. The fixed-effects component of each model included group (UBED vs. PELD), time (categorical: 3 days, 1 month, 6 months, and 12 months postoperatively), the corresponding preoperative baseline score, and group-by-time interaction. Wald chi-square tests were used to evaluate overall model effects and interactions.

When either the group main effect or the group-by-time interaction reached statistical significance, estimated marginal means were derived, and pairwise between-group comparisons at each follow-up time point were performed using Bonferroni correction for multiple comparisons. Between-group differences are reported as adjusted mean differences (AMDs) with 95% confidence intervals (CIs). To facilitate the interpretation of clinical significance, we referred to the established minimal clinically important difference (MCID) thresholds for VAS (1.5–2.0), ODI (10%–15%), and JOA scores (2, 3).

Hospitalization cost data were markedly skewed and are therefore reported as median (IQR), with between-group comparisons performed using Mann–Whitney *U* test. To identify independent predictors of total hospitalization cost, a generalized linear model (GLM) with a gamma distribution and a log link function was fitted. Candidate predictors included age, sex, BMI, symptom duration, preoperative ODI, VAS, JOA score, comorbidities, surgical procedure, operative level, stenosis subtype, operative time, intraoperative blood loss, length of total hospital stay, fluoroscopy times, and postoperative complications. Variables with *p* < 0.05 in univariable analysis were entered into multivariable model. Collinearity among covariates was assessed using the variance inflation factor (VIF), with VIF <5 considered acceptable. All tests were two-sided, and a *p* value <0.05 was considered statistically significant.

## Results

### Demographics and baseline characteristics

Table 1 summarizes the demographic and baseline characteristics of both groups. No statistically significant differences were observed between UBED and PELD groups in age, sex, BMI, comorbidities, symptom duration, surgical segments, stenosis subtype, preoperative ODI, VAS, or JOA score (*p* > 0.05). No significant between-group differences were observed at baseline, although small but clinically meaningful imbalances cannot be excluded given the sample size.

**Table 1:** Comparison of perioperative data between the two groups.

Variable	UBED (*n* = 42)	PELD (*n* = 52)	*t*/*χ*^2^/*Z*	*p* value
Gender			2.046	0.153
Male	24 (57.1)	22 (42.3)		
Female	18 (42.9)	30 (57.7)		
Age (years)	62.00 (54.75, 72.00)	67.00 (54.25, 72.50)	−0.175	0.861
BMI (kg/m^2^)	24.20 (22.67, 25.97)	23.27 (21.73, 26.25)	−0.696	0.486
Symptom duration (months)	12.00 (1.00, 92.75)	12.00 (1.25, 57.25)	−0.277	0.782
Comorbidity (yes)	24 (57.1)	30 (57.7)	0.003	0.957
Hypertension (yes)	18 (42.9)	26 (50.0)	0.476	0.490
Diabetes (yes)	7 (16.7)	9 (17.3)	0.007	0.934
Others (yes)	9 (21.4)	5 (9.6)	2.558	0.110
Surgical segment			5.078	0.139
L2/3	1 (2.4)	0 (0.0)		
L3/4	5 (11.9)	7 (13.5)		
L4/5	17 (40.5)	31 (59.6)		
L5/S1	19 (45.2)	14 (26.9)		
Stenosis subtype			0.190	0.979
Central	12 (28.6)	13 (25.0)		
Lateral recess	13 (31.0)	16 (30.8)		
Foraminal	11 (26.2)	15 (28.8)		
Mixed	6 (14.3)	8 (15.4)		
Preoperative VAS score (back pain)	6.00 (6.00, 7.00)	6.00 (6.00, 7.00)	−0.066	0.947
Preoperative VAS score (leg pain)	7.00 (7.00, 8.00)	7.00 (7.00, 8.00)	−0.301	0.763
Preoperative ODI	19.00 (16.00, 27.00)	22.00 (17.00, 29.00)	−0.898	0.369
Preoperative JOA score	10.00 (8.00, 12.00)	10.00 (8.00, 11.75)	−1.586	0.113
Operation time (min)	65.00 (50.00, 104.50)	73.00 (50.00, 100.00)	−0.221	0.825
Intraoperative blood loss (mL)	10.00 (5.00, 26.25)	5.00 (5.00, 10.00)	−3.035	0.002
Fluoroscopy times (count)	3.00 (2.00, 3.00)	4.00 (4.00, 5.00)	−6.695	<0.001
Ambulation time (hours)	12.50 (9.00, 15.25)	11.00 (9.00, 14.75)	−0.513	0.608
Hospital stay (days)				
Total	7.50 (5.00, 10.25)	7.00 (6.00, 10.00)	−0.107	0.915
Preoperative	2.00 (1.75, 3.00)	3.00 (2.00, 4.00)	−1.633	0.103
Postoperative	5.00 (3.00, 7.00)	4.00 (3.00, 6.00)	−1.162	0.245
Hospitalization expenses (×10^3^ RMB)				
Total	17.46 (15.38, 22.05)	15.82 (14.82, 17.38)	−2.525	0.012
Material	5.95 (5.63, 8.99)	5.74 (5.62, 5.98)	−2.186	0.029
Surgery	3.85 (3.85, 5.73)	3.85 (3.85, 3.85)	−3.183	0.001
Anesthesia	0.71 (0.49, 0.94)	0.49 (0.43, 0.52)	−4.162	<0.001
Medication	1.00 (0.59, 1.76)	0.98 (0.71, 1.35)	−0.578	0.563

UBED, unilateral biportal endoscopic decompression; PELD, percutaneous endoscopic lumbar decompression; BMI, body mass index; VAS, visual analogue scale; ODI, Oswestry disability index; JOA, Japanese Orthopaedic Association; RMB, Renminbi.

### Perioperative parameters

As shown in Table 1, intraoperative blood loss was significantly greater in UBED group than in PELD group (*p* = 0.002), whereas intraoperative fluoroscopy times was significantly less in UBED group (*p* < 0.001). No significant differences were detected between both groups in operative duration, time to ambulation, or length of hospital stay (*p* > 0.05).

### Clinical functional scores

Within-group pre- to postoperative comparisons: In both UBED and PELD groups, postoperative VAS scores for back pain, VAS scores for leg pain, ODI, and JOA scores at all time points improved significantly compared with preoperative baseline (*p* < 0.001), indicating that both techniques effectively relieved symptoms and restored function.

Between-group longitudinal comparisons: A GEE model, adjusted for the corresponding preoperative baseline score, was used to compare the two groups at each postoperative time point. The time effect was significant for all four outcomes (*p* < 0.001), indicating that scores continued to improve significantly from postoperative day 3 through the 12-month follow-up in both groups. Adjusted means, AMDs, and Bonferroni-corrected *p* values for between-group comparisons at each time point are presented in Table 2.

**Table 2:** Comparison of clinical outcome scores between the two groups.

Variable	Time point	Adjusted mean (95% CI)		Adjusted mean difference (95% CI)	Bonferroni *p* value
		UBED (*n* = 42)	PELD (*n* = 52)		
VAS score (back pain)	3 days	2.99 (2.87, 3.11)	3.12 (3.02, 3.22)	−0.13 (−0.38, 0.11)	>0.999
	1 month	2.18 (2.07, 2.29)	2.37 (2.26, 2.49)	−0.19 (−0.45, 0.06)	0.534
	6 months	1.78 (1.65, 1.90)	1.91 (1.81, 2.02)	−0.14 (−0.39, 0.12)	>0.999
	12 months	1.49 (1.36, 1.62)	1.66 (1.56, 1.76)	−0.17 (−0.44, 0.10)	>0.999
VAS score (leg pain)	3 days	3.00 (2.90, 3.10)	3.23 (3.12, 3.33)	−0.23 (−0.46, 0.01)	0.067
	1 month	2.05 (1.95, 2.15)	2.42 (2.32, 2.52)	−0.37 (−0.60, −0.15)	<0.001
	6 months	1.88 (1.76, 2.00)	2.00 (1.89, 2.10)	−0.11 (−0.37, 0.14)	>0.999
	12 months	1.69 (1.58, 1.80)	1.77 (1.69, 1.84)	−0.07 (−0.29, 0.14)	>0.999
ODI (%)	3 days	32.08 (31.80, 32.37)	32.59 (32.31, 32.86)	−0.51 (−1.14, 0.12)	0.336
	1 month	20.22 (19.99, 20.46)	22.57 (22.18, 22.96)	−2.35 (−3.06, −1.63)	<0.001
	6 months	18.34 (18.12, 18.57)	20.30 (19.92, 20.68)	−1.96 (−2.67, −1.25)	<0.001
	12 months	14.34 (14.00, 14.69)	15.01 (14.63, 15.39)	−0.67 (−1.49, 0.16)	0.315
JOA score	3 days	14.70 (14.40, 15.00)	15.32 (15.06, 15.57)	−0.62 (−1.24, 0.02)	0.064
	1 month	19.46 (19.33, 19.60)	18.61 (18.43, 18.78)	+0.86 (0.50, 1.22)	<0.001
	6 months	21.35 (21.16, 21.53)	20.91 (20.60, 21.22)	+0.43 (−0.14, 1.00)	0.505
	12 months	23.35 (23.18, 23.51)	22.89 (22.60, 23.19)	+0.45 (−0.08, 0.99)	0.238

UBED, unilateral biportal endoscopic decompression; PELD, percutaneous endoscopic lumbar decompression; VAS, visual analogue scale; ODI, Oswestry disability index; JOA, Japanese Orthopaedic Association; CI, confidence interval.

VAS scores for back pain: The group-by-time interaction was not significant (Wald *χ*^2^ = 0.527, *p* = 0.913), indicating parallel pain-relief trajectories between groups. Bonferroni-corrected *post-hoc* comparisons revealed no significant between-group differences in VAS scores for back pain at any time point (postoperative day 3, 1 month, 6 months, or 12 months; all adjusted *p* > 0.05). The AMDs ranged from −0.19 to −0.13 (UBED minus PELD), well below the established MCID for VAS score.

VAS scores for leg pain: The group main effect was significant (Wald *χ*^2^ = 14.128, *p* < 0.001), as was the group-by-time interaction (Wald *χ*^2^ = 11.489, *p* = 0.009), indicating different leg-pain relief trajectories between groups. Bonferroni-corrected *post-hoc* comparisons showed that VAS score for leg pain was significantly lower in UBED group than in PELD group at 1 month postoperatively (AMD = −0.37, 95% CI −0.60 to −0.15; adjusted *p* < 0.001), whereas no significant between-group differences were observed at postoperative day 3, 6 months, or 12 months (all adjusted *p* > 0.05). The maximum between-group difference of 0.37 points represented only approximately 25% of the MCID for VAS score (approximately 1.5).

ODI: The group main effect was significant (Wald *χ*^2^ = 68.711, *p* < 0.001), as was the group-by-time interaction (Wald *χ*^2^ = 35.332, *p* < 0.001). Bonferroni-corrected *post-hoc* comparisons demonstrated ODI was significantly lower in UBED group than in PELD group at both 1 month (AMD = −2.35, 95% CI −3.06 to −1.63; adjusted *p* < 0.001) and 6 months postoperatively (AMD = −1.96, 95% CI −2.67 to −1.25; adjusted *p* < 0.001). No significant between-group differences were observed at postoperative day 3 or 12 months (both adjusted *p* > 0.05). The maximum between-group difference of 2.35 represented approximately 25% of the established MCID for ODI (approximately 10).

JOA scores: The group main effect was significant (Wald *χ*^2^ = 5.494, *p* = 0.019), as was the group-by-time interaction (Wald *χ*^2^ = 64.494, *p* < 0.001). Bonferroni-corrected *post-hoc* comparisons revealed JOA score was significantly higher in UBED group than in PELD group at 1 month postoperatively (AMD = 0.86, 95% CI 0.50–1.22; adjusted *p* < 0.001), whereas no significant between-group differences were observed at postoperative day 3, 6 months, or 12 months (all adjusted *p* > 0.05). The maximum between-group difference of 0.86 points (on a 0–29 scale) was below the reported MCID of approximately 2–3 points for JOA score.

### Clinical efficacy assessment

According to modified MacNab criteria at 12 months after surgery, the excellent-good rate of UBED group was 97.6% (30 excellent, 11 good, 1 fair), and that of the PELD group was 84.6% (33 excellent, 11 good, 6 fair, 2 poor). According to Table 3, efficacy differences based on modified MacNab criteria were statistically significant between both groups (*p* = 0.039).

**Table 3:** Comparison of clinical efficacy and complications between the two groups.

Variable	UBED (*n* = 42)	PELD (*n* = 52)	*t*/*χ*^2^/*Z*	*p* value
Clinical efficacy			NR	0.039
Excellent + good	41 (97.6)	44 (84.6)		
Fair + poor	1 (2.4)	8 (15.4)		
Complications (yes)	2 (4.8)	10 (19.2)	4.368	0.037
Transient leg numbness (yes)	1 (2.4)	6 (11.5)	NR	0.126
Recurrence (yes)	1 (2.4)	3 (5.8)	NR	0.626
Infection (yes)	0 (0)	1 (1.9)	NR	>0.999
Reoperation (yes)	0 (0)	3 (5.8)	NR	0.251

UBED, unilateral biportal endoscopic decompression; PELD, percutaneous endoscopic lumbar decompression; NR, not reported, meaning that Fisher's exact test was employed.

### Complications

The overall complication rate was significantly lower in UBED group (4.8%) than in PELD group (19.2%) (*p* = 0.037). In PELD group, three cases of recurrence were observed, all of which resolved following reoperation; the corresponding reoperation rate was 5.8%. No statistically significant difference in reoperation rate was observed between both groups (*p* = 0.251). Table 3 details the complications and reoperations.

### Cost analysis

The between-group comparison of hospitalization costs is summarized in Table 1. Total hospitalization cost was significantly higher in UBED group than in PELD group [17.46 (15.38, 22.05) vs. 15.82 (14.82, 17.38); *Z* = −2.525, *p* = 0.012]. Component-wise analysis showed that surgical expense [3.85 (3.85, 5.73) vs. 3.85 (3.85, 3.85); *Z* = −3.183, *p* = 0.001], anesthesia expense [0.71 (0.49, 0.94) vs. 0.49 (0.43, 0.52); *Z* = −4.162, *p* < 0.001], and material cost [5.95 (5.63, 8.99) vs. 5.74 (5.62, 5.98); *Z* = −2.186, *p* = 0.029] were all significantly higher in UBED group than in PELD group, whereas medication cost did not differ significantly between groups [1.00 (0.59, 1.76) vs. 0.98 (0.71, 1.35); *Z* = −0.578, *p* = 0.563].

Univariate analysis identified symptom duration, operative time, intraoperative blood loss, length of total hospital stay, preoperative ODI, preoperative leg VAS score, comorbidities, and surgical type as factors significantly associated with total hospitalization cost (*p* < 0.05). Due to substantial multicollinearity, preoperative ODI and leg VAS score were excluded from subsequent modeling. To identify independent predictors of total hospitalization cost, a GLM with a gamma distribution and log link was constructed, using total hospitalization cost as the dependent variable and operation type, symptom duration, number of comorbidities, operation time, intraoperative blood loss, and total hospital stay as covariates. Final GLM showed an acceptable fit (scaled deviance/df = 1.08; scaled Pearson *χ*^2^/df = 1.12), and all VIFs were below 1.6 (range 1.022–1.588), indicating no significant multicollinearity. Operation type [exp(B) = 0.917, 95% CI (0.877, 0.959), *p* < 0.001], total hospital stay [exp(B) = 1.037, 95% CI (1.029, 1.045), *p* < 0.001], and intraoperative blood loss [exp(B) = 1.001, 95% CI (1.000, 1.002), *p* = 0.027] were independently associated with total hospitalization cost. Detailed information is recorded in Supplementary File 2.

## Discussion

This study adopted a single-center, retrospective cohort design to comparatively assess perioperative parameters, clinical functional improvement, complications, and hospital-related medical costs, between UBED and PELD for single-level DLSS. Our results showed that UBED was associated with greater blood loss but significantly fewer fluoroscopic exposures compared with PELD. No statistically significant differences were observed in operative duration, time to postoperative ambulation, or length of hospital stay. Both techniques demonstrated robust efficacy in alleviating back and leg pain and improving lumbar function. UBED group demonstrated slightly better early postoperative outcomes, with lower leg pain VAS scores and ODI and higher JOA scores at 1 month, as well as a higher 12-month excellent-good rate. Furthermore, UBED was associated with a lower overall complication rate. However, total hospitalization cost, surgical cost, anesthesia cost and material cost were all significantly higher in UBED group than in PELD group. Multivariate regression analysis confirmed that surgical technique, length of hospital stay, and intraoperative blood loss were independent predictors of total hospital medical expenditure.

From a perioperative standpoint, the two procedures exhibited distinct technical profiles, attributable to fundamental differences in their surgical approaches and procedural logic. Consistent with this, intraoperative blood loss was significantly greater in UBED group relative to PELD group. The underlying mechanism was directly linked to the decompression extent and the degree of surgical approach-related trauma. In UBED, two channels were established beside the posterior midline, necessitating moderate blunt dissection of the paravertebral musculature. Decompression encompassed the central spinal canal and lateral recesses, entailing a broader bony resection, thereby increasing the likelihood of epidural venous plexus exposure and associated injury risk. In contrast, PELD utilized a single working channel introduced through a transforaminal lateral approach, with minimal to no dissection of paravertebral soft tissues. Bony intervention was confined strictly to the intervertebral foramen region, resulting in substantially lower overall tissue trauma and reduced intraoperative blood loss (17). This advantage is especially critical for elderly or medically frail patients. As described in the long-term findings by Houra et al., PELD represented a safe and effective option for single-segment spinal stenosis, characterized by minimal blood loss and feasibility under local anesthesia, conferring a distinct clinical advantage over UBED in patients with significant comorbidities (20). It should be acknowledged that estimated blood loss in endoscopic procedures is an indirect measure based on suction and irrigation volumes, and may not be as precise as in open surgery. Nevertheless, because the same measurement protocol was applied to both groups, the between-group comparison remains valid.

Regarding fluoroscopic exposure, intraoperative fluoroscopy times were significantly fewer in UBED group compared with PELD group. The posterior approach employed in UBED offers well-defined surface anatomical landmarks. Once the working channel is established, key osseous landmarks, including spinous processes and laminae, are readily identifiable under endoscopic visualization, enabling predominantly direct-vision-guided surgery without repeated fluoroscopic confirmation. Conversely, PELD relies heavily on real-time imaging guidance for both initial needle puncture and subsequent foraminoplasty. Precise positioning and angulation of the working cannula require iterative fluoroscopic adjustments to avoid iatrogenic nerve root injury, consequently prolonging radiation exposure (18, 21). It is noteworthy that although intraoperative bleeding was slightly higher in UBED group, no statistically significant differences were observed between both groups in postoperative ambulation time or length of hospital stay. This suggests that UBED did not impede early postoperative recovery despite the modest increase in bleeding, and its minimally invasive profile remains comparable to that of PELD.

At the level of functional recovery, the present study demonstrates that both minimally invasive decompression techniques continuously improve pain symptom and physical function. Postoperative ODI, VAS, and JOA scores at all time points were significantly improved relative to preoperative baseline in both groups. Using a GEE model adjusted for the corresponding preoperative baseline score, we longitudinally compared postoperative functional outcomes between UBED and PELD for single-level DLSS. Overall, the two techniques yielded comparable postoperative back-pain relief. For indices directly reflecting nerve-root decompression, including VAS scores for leg pain, ODI, and JOA scores, UBED group showed a slight early advantage at approximately 1 month postoperatively, but this advantage gradually diminished and had essentially converged between groups by the 12-month follow-up. VAS score for back pain did not differ significantly between groups at any of the four postoperative time points, with between-group differences of only 0.13–0.19 points, well below the established MCID. This finding suggests that, despite the biportal approach of UBED and its potentially slightly greater exposure of the paraspinal muscles, the degree of disruption to the posterior muscular-ligamentous complex was limited with both minimally invasive techniques, resulting in no substantive difference in postoperative back-pain relief. This is consistent with the technical feature that both approaches traverse a muscle-sparing interval without extensive dissection or disruption of the paraspinal muscles and supraspinous ligament. In contrast to back pain, VAS score for leg pain, ODI, and JOA score, three indices reflecting nerve decompression, all revealed a slight early advantage in UBED group. The mechanism underlying this observation may be that the dual-channel design of UBED separates the viewing and working portals, allowing the surgeon to use conventional open instruments under a more expansive direct view to achieve more thorough bony decompression of the central canal and lateral recess, thereby producing more complete nerve-root release and consequently faster early relief of radicular pain and functional recovery (17, 22–24). The between-group difference in ODI persisted significantly at both 1 and 6 months, longer than the corresponding differences in VAS score for leg pain and JOA score. This may be because ODI comprehensively captures multiple daily functions such as walking, standing, and sleeping, and is therefore more sensitive to variations in the thoroughness of decompression.

Nevertheless, the clinical significance of these statistical differences requires cautious interpretation. The maximum between-group differences across the four outcomes were 0.37 points for leg VAS score, 2.35 points for ODI, and 0.86 points for JOA score, all of which fell below the established MCID for the respective instruments. This indicates that, although the GEE model detected statistically significant differences owing to its high statistical power, patients themselves would be unlikely to perceive such small differences in daily life. Furthermore, no between-group differences remained significant at 12 months, and the long-term functional scores of the two groups nearly overlapped, suggesting that as nerve-root edema resolves and neural recovery proceeds, both techniques ultimately achieve adequate neurological recovery, and the early advantage of UBED does not translate into sustained long-term benefit. This observation is consistent with the findings of several comparative studies (25). However, based on modified MacNab criteria, UBED group achieved the excellent-good rate of 97.6%, significantly higher than that of 84.6% observed in PELD group, further indicating that the better early pain relief and functional recovery can yield higher patient satisfaction and overall clinical efficacy, an advantage that appears to be particularly pronounced in cases with more severe stenosis. The slightly better early postoperative outcomes observed in UBED group may be related to the technical characteristics of the dual-channel approach, which allows separate viewing and working portals and may facilitate more extensive bony removal of the lateral recess and intervertebral foramen under direct visualization. However, this is a proposed mechanism rather than a demonstrated finding, as no postoperative radiological measurements of decompression adequacy were obtained in this study. Future studies with postoperative MRI or CT measurements of dural sac cross-sectional area, foraminal diameter, and residual stenosis are needed to verify whether the theoretical advantages of UBED translate into objectively greater decompression.

Complication rates and reoperation risk constitute core safety endpoints in evaluating surgical interventions. In this study, the overall complication rate was significantly lower in UBED group (4.8%) compared with PELD group (19.2%). This numerically lower complication rate in UBED can be attributed, at least in part, to the fundamental design principle of UBED: the spatial separation of visualization and instrumentation channels. This configuration affords an unobstructed, high-definition surgical field and markedly enhances instrument maneuverability, thereby enabling precise, direct-vision dissection of hyperplastic tissue and targeted resection of hypertrophic ligamentum flavum or osteophytes. As a result, the dual-channel design may theoretically reduce the risk of iatrogenic nerve root traction injury and dural tear (26, 27). On the other hand, PELD imposes inherent limitations related to operative angulation, resulting in a blind zone, particularly when managing thickened ligamentum flavum or bony hyperplasia. Moreover, during transforaminal foraminoplasty, instrument manipulation occurs in close proximity to the nerve root, thereby increasing the risk of iatrogenic nerve stimulation or injury (28). Regarding reoperation rates, three patients in PELD group experienced postoperative symptom recurrence and subsequently underwent secondary surgery, yielding a reoperation rate of 5.8%. However, this difference did not reach statistical significance between both groups. The marginally higher recurrence risk observed in PELD group may be attributable to incomplete neural decompression, residual disc material, or recurrent bony overgrowth leading to renewed nerve compression (29, 30). Nevertheless, given the study's limited sample size and one-year follow-up duration, long-term differences in recurrence rates warrant further validation through extended longitudinal observation.

Cost analysis serves as a critical complement to this study, offering an evidence-based economic foundation for optimizing healthcare resource allocation and informing clinical decision-making. The present study provided a detailed breakdown of hospitalization costs between the two techniques. The total hospitalization cost was significantly higher in UBED group than in PELD group (17.46 vs. 15.82; *p* = 0.012). This difference should not be attributed to a single factor but reflects the combined effect of multiple technique-related cost components. Component-wise analysis revealed that this difference was driven primarily by two factors. Firstly, material cost was significantly higher in UBED group (5.95 vs. 5.74; *p* = 0.029), which is attributable to the dual-channel endoscopic instruments, bipolar radiofrequency systems, and more frequent use of drill-based bony decompression required in UBED (31). Secondly, UBED was performed under general anesthesia, resulting in significantly higher anesthesia expense than PELD performed under local anesthesia with sedation (0.71 vs. 0.49; *p* < 0.001). In contrast, PELD employs a single-channel endoscopic platform characterized by a simpler consumable architecture and correspondingly lower associated costs (9). Notably, medication cost did not differ significantly between groups (1.00 vs. 0.98; *p* = 0.563), indicating that postoperative analgesia and routine medication regimens were comparable. In addition, routine closed-suction drain placement and more frequent use of hemostatic materials in UBED further contributed to surgical and material expenses. Multivariate analysis using a GLM identified surgical approach, length of hospital stay, and intraoperative blood loss as independent predictors of total hospitalization cost. Among them, the cost differential attributable to the surgical approach itself constitutes the primary driver of overall cost variation. After adjusting for other covariates, the total cost in PELD group was approximately 91.7% of that in UBED group. Each additional day of hospitalization was associated with an approximate 3.7% increase in total hospitalization cost, highlighting shortened length of stay as a pivotal lever for cost containment, a finding highly aligned with the cost-control objectives of enhanced recovery after surgery. Similarly, each 10 mL increment in intraoperative blood loss corresponded to an approximate 1.0% rise in total hospitalization cost. Blood loss was independently associated with total hospitalization cost in the GLM. However, this should be interpreted as an association rather than a causal effect, as blood loss may correlate with operative complexity, procedure duration, and other unmeasured factors that may independently influence cost. It should be clarified that the four reported categories, such as surgical, anesthesia, material, and medication expenses, represent the principal technique-related cost components. The remaining portion of total hospitalization cost consists primarily of fixed overhead items, including bed fees, nursing fees, routine laboratory tests, and postoperative imaging, which are determined mainly by length of hospital stay rather than by the surgical approach. Because total length of stay did not differ significantly between groups, these fixed items were not separately itemized. The analysis encompasses only direct medical costs incurred during the index admission, and capital depreciation of reusable equipment (e.g., endoscopic towers, C-arm fluoroscopy systems) is not included. The cost data were derived from a single center under a specific regional reimbursement system, and variations in centralized procurement, anesthesia pricing, and hospital reimbursement across institutions may limit the generalizability of our findings.

This study offers several methodological strengths. Firstly, it simultaneously evaluated both clinical outcomes and hospitalization costs, providing a more comprehensive basis for surgical decision-making than studies focusing solely on functional outcomes. Secondly, all procedures were performed by a single experienced surgeon, minimizing inter-surgeon variability. Thirdly, the GEE model was adopted to fully utilize the repeated measurement data, controlling for baseline confounding factors, and the statistical analysis method was relatively rigorous. Fourthly, recognizing the inherently right-skewed distribution of medical cost data, we employed a GLM with a gamma distribution and log-link function for multivariate analysis. This statistical approach is theoretically appropriate for cost data, thereby enhancing both the validity and reliability of the analytical results.

However, this study has several inherent limitations. Firstly, it employed a single-center, retrospective design. Surgical modality was determined jointly by the treating physician and the patient, not through random allocation, thereby introducing inevitable selection bias. Consequently, control of baseline confounding factors was less robust than in randomized controlled trials. Although no baseline characteristic differed significantly between groups, the absence of statistical significance does not imply equivalence, and clinically meaningful residual confounding cannot be excluded. UBED group may have more severe central or lateral recess stenosis at baseline. To mitigate this, we adjusted for preoperative baseline scores in all repeated-measures outcome models. Because this was a purely retrospective study, VAS, ODI, and JOA scores were obtained from patient recall at the time of data collection. Although available outpatient records were used to corroborate these scores, recall bias cannot be excluded. Future prospective studies with standardized, real-time outcome documentation are warranted to confirm our findings. Secondly, the sample size was relatively modest, limiting statistical power to detect low-frequency outcomes such as complications and reoperations. This might compromise the reliability and generalizability of the findings. Thirdly, the follow-up period was restricted to 12 months, precluding assessment of long-term efficacy, recurrence rates, and cost-effectiveness beyond 2 years. Fourthly, the two techniques were performed under different anesthesia modalities (general anesthesia for UBED vs. local anesthesia for PELD), which is an inherent feature of each approach and cannot be separated in a non-randomized comparison. This difference may independently influence hospitalization cost, postoperative recovery, and complication rates. Future comparative studies should attempt to disentangle the effect of the decompression technique from that of the anesthesia modality, for example by comparing UBED performed under local anesthesia or by including anesthesia-related covariates in adjusted analyses. Fifthly, this study did not include postoperative radiological measurements (e.g., dural sac cross-sectional area, foraminal diameter). Therefore, claims regarding the extent or quality of decompression cannot be objectively verified. The slightly better early clinical outcomes in UBED group should not be interpreted as definitive evidence of superior decompression and radiological outcome studies are needed to confirm this. Furthermore, only direct hospitalization medical costs were included while indirect costs such as postoperative rehabilitation, outpatient follow-up, and productivity loss were not captured. Cost figures reflect local reimbursement policies and implant pricing at our institution, limiting generalizability to other settings. No utility measures (e.g., EQ-5D) or longer-term outcomes were collected, so the present cost comparison should not be interpreted as a formal cost-effectiveness analysis. Therefore, future multicenter studies with longer follow-up, direct utility measurement, and a broader societal cost perspective are needed to fully evaluate the health economics of these two techniques.

## Conclusion

This single-center retrospective study compared perioperative outcomes, clinical functional results, complications, and hospitalization costs of UBED vs. PELD for single-segment DLSS. Both techniques achieved significant and sustained improvements in pain, function, and neurological status over the 12-month follow-up period. UBED group showed fewer intraoperative fluoroscopy times, modestly better early postoperative outcomes at 1 month, a higher 12-month excellent-good rate, and a numerically lower complication rate. However, UBED was associated with greater intraoperative blood loss and higher total hospitalization costs. No significant between-group differences were found in operative time, ambulation time, or length of hospital stay. In the cost analysis, total hospitalization cost, surgical cost, anesthesia cost and material cost were all higher in UBED group. Surgical approach, hospital stay, and intraoperative blood loss were independent predictors of total hospitalization cost. PELD was associated with lower hospitalization costs. In clinical practice, the selection of operative strategy should be individualized, with consideration given to both the patient's clinical characteristics and socioeconomic circumstances, to optimize the balance between therapeutic benefit and healthcare resource utilization.

## Data Availability

The raw data supporting the conclusions of this article will be made available by the authors, without undue reservation.
