# Supplementary material for: Comparison of unilateral biportal endoscopic decompression and percutaneous endoscopic lumbar decompression for single-segment degenerative lumbar spinal stenosis: a retrospective comparative study involving clinical efficacy and cost analysis
Source: Front Med (Lausanne). 2026 Oct 26;13:1978780. doi: 10.3389/fmed.2026.1978780 (PMC13745268; doi:10.3389/fmed.2026.1978780)
Supplement: Supplementary file 1 [file Datasheet1.pdf]

**Surgical procedure**

**UBED Group**

After endotracheal intubation and general anesthesia, patients were positioned prone. Under fluoroscopic guidance to confirm the target level, two horizontal incision lines were drawn approximately 1.0–1.5 cm above and below the skin projection of the affected disc space. The viewing portal was placed on the surgeon's left side with a skin incision of approximately 1 cm, and the working portal was placed on the surgeon's right side with a skin incision of approximately 1.5 cm.

After routine disinfection and draping, transverse skin incisions were made along the marked lines, and the fascia was incised longitudinally. Primary dilators were inserted through both portals; after fluoroscopic confirmation of adequate positioning, serial dilatation was performed. An endoscope was introduced through the viewing portal for visualization and continuous irrigation, while a plasma radiofrequency probe was introduced through the working portal. Soft tissue at the junction of the vertebral lamina and spinous process was cleared to expose the lower border of the superior lamina, the upper border of the inferior lamina, the ligamentum flavum, and the medial aspect of the zygapophysial joints.

A high-speed drill was used to thin the upper border of the inferior lamina, the lower border of the superior lamina, and the medial one-third of the facet joint. Kerrison rongeurs were then used to remove bone up to the origin of the ligamentum flavum on the superior lamina, its insertion on the inferior lamina, and the medial one-third of the facet joint. The ligamentum flavum was then dissected, stripped, and removed to expose the ipsilateral dural sac. Decompression was performed along the lateral aspect of the dural sac and nerve root until the nerve root was fully exposed.

If disc herniation was present, free nucleus pulposus was removed. If contralateral decompression was required, a high-speed drill was used at the junction of the superior lamina and spinous process to resect the lower border of the contralateral lamina and the medial aspect of the contralateral facet joint, working on the surface of the contralateral ligamentum flavum. The contralateral ligamentum flavum was then removed, and the contralateral dura and nerve root were identified and protected. The medial border of the contralateral superior articular process and the upper border of the contralateral lamina were then removed to enlarge the contralateral lateral recess. After meticulous hemostasis under endoscopic visualization and confirmation of no active bleeding, instruments were withdrawn. A closed suction drain was placed through the working portal, and the incisions were closed routinely.

**PELD Group**

Patients were positioned prone on a radiolucent table, with chest bolsters placed to keep the abdomen free of compression. After fluoroscopic identification of the target level, the puncture point was selected approximately 8–14 cm lateral to the midline, adjusted according to body habitus and local soft-tissue thickness. Local infiltration anesthesia was applied to the subcutaneous tissue, deep fascia, and periarticular region. Under fluoroscopic guidance, the puncture needle was advanced until its tip reached the target area: on the anteroposterior view, the tip should lie at the lateral border of the superior articular process of the lower vertebral body; on the lateral view, at the ventral aspect of the superior articular process.

A guidewire was then inserted, a 0.8-cm skin incision was made, and serial dilators were advanced along the guidewire. The working cannula was positioned and connected to the endoscopic system. Radiofrequency ablation was used to clear soft tissue around the superior articular process and to clearly identify its bony margin. Under endoscopic visualization, a visual trephine was used to resect the anterior portion of the superior articular process of the lower vertebral body, thereby enlarging the intervertebral foramen and lateral recess and relieving bony stenosis.

The posterior longitudinal ligament and ligamentum flavum were then removed to decompress the nerve root, with decompression performed both ventrally and dorsally to the dural sac. Discectomy was performed selectively according to the degree of disc herniation: herniated nucleus pulposus tissue was removed, and residual disc fragments were explored and cleared to achieve adequate decompression. After endoscopic confirmation that the dura had resumed pulsation and that the straight leg-raising test was negative, radiofrequency hemostasis were performed. The endoscope was then withdrawn, and the incision was closed (Figure S1).

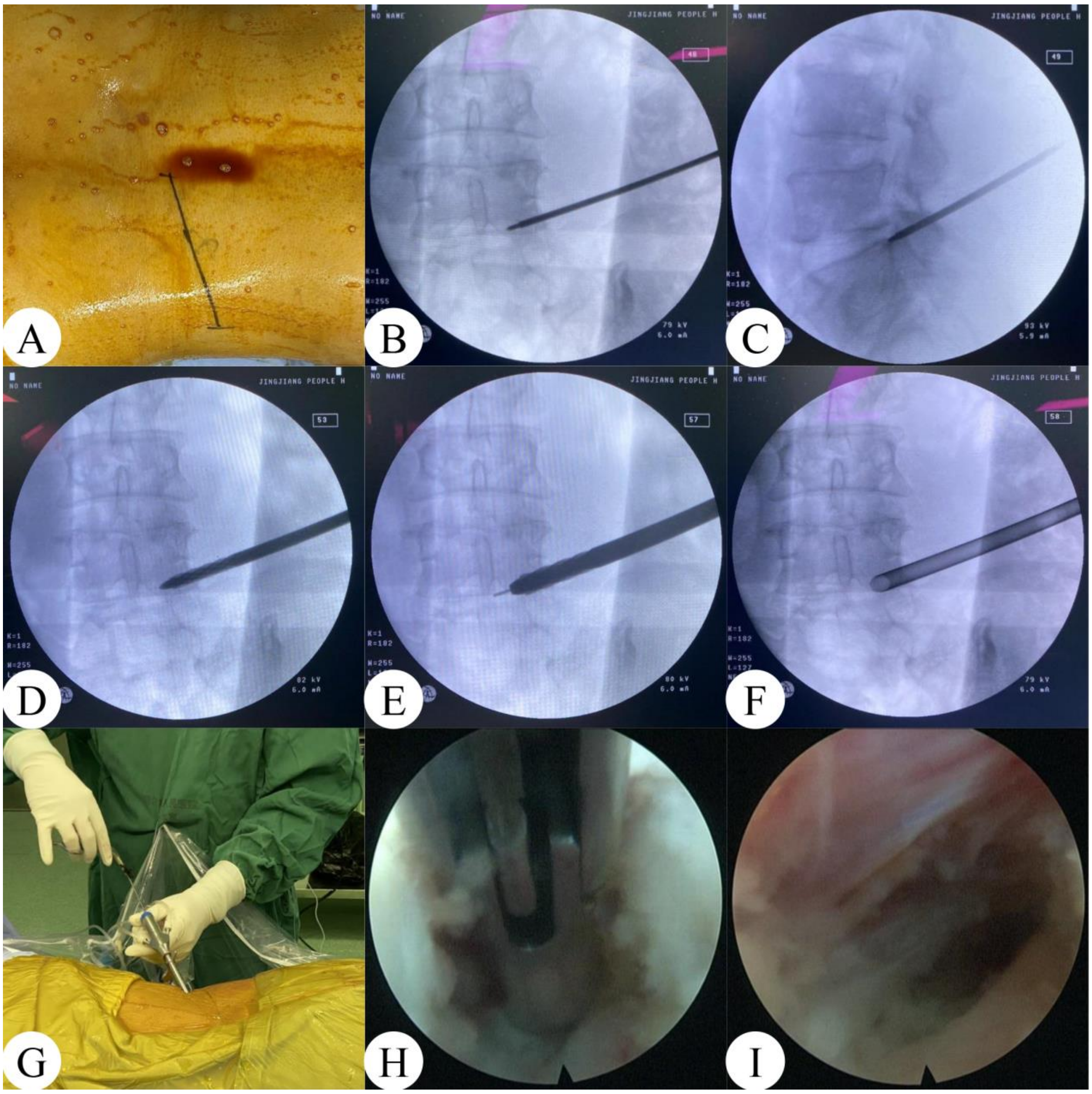


Figure S1 PELD for L4/5 spinal canal stenosis. (A-C) The puncture needle reaches the ventral aspect of the L5 superior articular process; (D,E) Sequential dilation of the soft tissue is performed using sleeves; (F) The working cannula is placed; (G) The endoscopic system is connected; (H) The spinal canal is decompressed; (I) Nerve root relaxation is confirmed upon exploration.

**Surgeon experience and technique selection**

All procedures were performed by a single senior spine surgeon with more than 5 years of experience in endoscopic spine surgery. Before patient enrollment, this surgeon had already completed the formal learning curves for both UBED and PELD. Therefore, all included patients were treated during the surgeon's stable, experienced phase, minimizing performance bias.

The choice between UBED and PELD was determined preoperatively by the same attending surgeon based on patient-specific factors and lesion characteristics:
UBED was generally preferred for central canal stenosis, lateral recess stenosis, or combined central/lateral recess stenosis in patients suitable for general anesthesia.
PELD (transforaminal) was generally preferred for isolated foraminal stenosis, as well as for elderly patients or those with high anesthetic risk, given that PELD can be safely performed under local anesthesia.

**Standardized blood loss measurement method**

Intraoperative blood loss was estimated using a standardized protocol. At the conclusion of each procedure, the total volume of fluid collected in the suction canister was measured, and the total volume of irrigation normal saline used during the procedure was recorded. Estimated intraoperative blood loss was calculated as the difference between total suction volume and total irrigation volume. The weight difference of surgical gauzes before and after use (1 g ≈ 1 mL) was also added. The same measurement protocol was applied to both the UBED and PELD groups.

**Postoperative discharge criteria and drain-removal protocol**

Postoperatively, a closed suction drain was routinely placed in the UBED group and removed when the drainage volume was less than 30 mL in 24 hours (typically 48–72 hours after surgery). In the PELD group performed under local anesthesia, no drain was placed.

Discharge was considered when the following criteria were met: (1) afebrile with no wound erythema or drainage; (2) ability to ambulate independently with or without a lumbar brace; (3) adequate pain control with oral analgesics; (4) no neurological deterioration; and (5) stable postoperative imaging findings if indicated.
