# Supplementary material for: Comparison of unilateral biportal endoscopic decompression and percutaneous endoscopic lumbar decompression for single-segment degenerative lumbar spinal stenosis: a retrospective comparative study involving clinical efficacy and cost analysis
Source: Front Med (Lausanne). 2026 Oct 26;13:1978780. doi: 10.3389/fmed.2026.1978780 (PMC13745268; doi:10.3389/fmed.2026.1978780)
Supplement: Supplementary file 2 [file Datasheet2.pdf]

Table S1 Results of univariate analysis

Variable	Correlation coefficient	P value
Age	0.183	0.078
Body mass index	0.124	0.234
Symptom duration	0.217	0.036
Operation time	0.258	0.012
Intraoperative blood loss	0.490	< 0.001
Total hospital stay	0.770	< 0.001
Fluoroscopy times	-0.125	0.231
Preoperative back VAS score	0.179	0.085
Preoperative leg VAS score	0.229	0.027
Preoperative ODI	0.226	0.028
Preoperative JOA score	0.203	0.050
Comorbidities	0.215	0.037
Operation type	-0.262	0.011
Sex	-0.09	0.387
Surgical segment	0.07	0.503
Stenosis subtype	0.098	0.346
Complication	-0.132	0.206

VAS, visual analogue scale; ODI, Oswestry disability index; JOA, Japanese Orthopaedic Association.

Table S2 Results of multivariate analysis

Parameter	Exp(B)	95% Wald Confidence Interval	Wald χ²	P value	VIF
Operation type (PELD vs. UBED)	0.917	0.877–0.959	14.828	<0.001	1.110
Total hospital stay (per day)	1.037	1.029–1.045	103.435	<0.001	1.092
Intraoperative blood loss (per mL)	1.001	1.000–1.002	4.899	0.027	1.588
Comorbidities (per unit)	1.019	0.993–1.046	2.029	0.154	1.063
Symptom duration (per month)	1.000	1.000–1.001	2.453	0.117	1.022
Operation time (per min)	1.000	1.000–1.001	1.029	0.310	1.497

UBED, unilateral biportal endoscopic decompression; PELD, percutaneous endoscopic lumbar decompression; VIF, variance inflation factor.

Table S3 Goodness-of-fit statistics

Fit measure	Value
Scaled deviance / df	1.08
Scaled Pearson χ² / df	1.12
Log likelihood	−835.949
AIC	1687.900
BIC	1708.240

AIC, Akaike Information Criterion; BIC, Bayesian Information Criterion.

Table S4 Final GLM predicting total hospitalization cost

Parameter	Coding	Reference category
Operation type	1 = UBED, 2 = PELD	UBED (group=1)
Symptom duration	Continuous (months)	—
Comorbidities	Continuous (count)	—
Operation time	Continuous (min)	—
Intraoperative blood loss	Continuous (mL)	—
Total hospital stay	Continuous (days)	—

UBED, unilateral biportal endoscopic decompression; PELD, percutaneous endoscopic lumbar decompression.
